# A benign juvenile environment reduces the strength of antagonistic pleiotropy and genetic variation in the rate of senescence

**DOI:** 10.1111/1365-2656.12468

**Published:** 2015-12-19

**Authors:** Sin‐Yeon Kim, Neil B. Metcalfe, Alberto Velando

**Affiliations:** ^1^ Departamento de Ecoloxía e Bioloxía AnimalUniversidade de VigoVigo36310Spain; ^2^ Institute of Biodiversity, Animal Health and Comparative MedicineCollege of Medical, Veterinary and Life SciencesUniversity of GlasgowGlasgowG12 8QQUK

**Keywords:** animal model, disposable soma, G × E, phenotypic plasticity, random regression, trade‐off

## Abstract

1. The environment can play an important role in the evolution of senescence because the optimal allocation between somatic maintenance and reproduction depends on external factors influencing life expectancy.
2. The aims of this study were to experimentally test whether environmental conditions during early life can shape senescence schedules, and if so, to examine whether variation among individuals or genotypes with respect to the degree of ageing differs across environments.
3. We tested life‐history plasticity and quantified genetic effects on the pattern of senescence across different environments within a reaction norm framework by using an experiment on the three‐spined stickleback (*Gasterosteus aculeatus*, Linnaeus) in which F1 families originating from a wild annual population experienced different temperature regimes.
4. Male sticklebacks that had experienced a more benign environment earlier in life subsequently reduced their investment in carotenoid‐based sexual signals early in the breeding season, and consequently senesced at a slower rate later in the season, compared to those that had developed under harsher conditions. This plasticity of ageing was genetically determined. Both antagonistic pleiotropy and genetic variation in the rate of senescence were evident only in the individuals raised in the harsher environment.
5. The experimental demonstration of genotype‐by‐environment interactions influencing the rate of reproductive senescence provides interesting insights into the role of the environment in the evolution of life histories. The results suggest that benign conditions weaken the scope for senescence to evolve and that the dependence on the environment may maintain genetic variation under selection.

## Introduction

The ability of a genotype to produce different phenotypes in response to distinct environmental conditions (‘phenotypic plasticity’, Pigliucci 2001) has a profound importance in the evolution of life histories. Under the presence of within‐population variation in the extent of life‐history plasticity among genotypes, selection can shape an optimal response to the environmental gradient (‘reaction norm’), and this may enable organisms to maximize fitness by optimizing their life‐history phenotype (Stearns 1992; Flatt & Heyland 2011). An increasing number of studies have reported plastic phenological responses of animals to climate change (reviewed in Charmantier & Gienapp 2014; Merila & Hendry 2014), and some have studied how individuals or populations vary with respect to the degree of plasticity (e.g. Husby *et al*. 2010). Life‐history theory predicts that the linkages among traits should also change across environments if the degree of plasticity varies among individuals, since individual variation in energy allocation can determine the pattern of phenotypic relation among traits (Stearns 1992).

Senescence, the decline in physiological condition and performance with age, is assumed to occur as a result of an accumulation of late‐acting deleterious mutations (Medawar 1952), a trade‐off between reproduction and cellular maintenance (disposable soma theory, Kirkwood 1977) and active selection of alleles with beneficial effects in early life but pleiotropic deleterious effects in late life (antagonistic pleiotropy theory, Williams 1957). In previous studies of these theories of ageing, the effect of reproductive effort on the rate of senescence was consistent with the resource allocation principles underlying the disposable soma theory (e.g. Nussey *et al*. 2006; Kim *et al*. 2011; Boonekamp *et al*. 2014), while genetic correlations between early and late performances supported the antagonistic pleiotropic theory (e.g. Partridge, Prowse & Pignatelli 1999; Charmantier *et al*. 2006). However, some studies failed to detect these patterns probably due to natural variation among individuals in genetic quality and/or plastic responses of genotypes to environmental conditions (Lemaître *et al*. 2015). Although some recent studies have recognized the existence of within‐ and among‐population variation in the patterns of senescence, the causes of such variation remain poorly understood (Wilson, Charmantier & Hadfield 2008; Bouwhuis *et al*. 2010; Nussey *et al*. 2013; Jones *et al*. 2014).

Changes in the external environment may dynamically alter the optimal investment in somatic maintenance and reproduction, and thereby promote or hinder the evolution of senescence (Kirkwood 2002; Reznick *et al*. 2004). These environmental effects are not restricted to the period after sexual maturation, since conditions experienced earlier in life can alter the optimal schedule of reproduction, especially if they influence developmental pathways (West‐Eberhard 2003; Lemaître *et al*. 2015). If environmental conditions encountered during development cause a reduced investment in early reproduction, then it is predicted that the level of somatic damage or strength of antagonistic pleiotropy will also be reduced, thereby decelerating the rate of ageing (McNamara *et al*. 2009). Indeed, a recent study demonstrated that non‐genetic maternal effects on growth and maturation consequently modulated the patterns of senescence and mortality in *Daphnia pulex* (Plaistow *et al*. 2015). So can environmental conditions shape senescence schedules? This question may be best approached by simultaneously testing plasticity of ageing and quantifying genetic effects on the pattern of senescence across different environments within a reaction norm framework (Nussey, Wilson & Brommer 2007; Monaghan *et al*. 2008; Wilson, Charmantier & Hadfield 2008; Charmantier, Brommer & Nussey 2014).

Here, we studied plasticity of ageing patterns in response to environmental conditions by using an experiment on the three‐spined stickleback in which F1 families originating from a wild annual population experienced different temperature regimes over the winter prior to the reproductive season. By manipulating an environmental factor other than resources, we tested the ability of different genotypes to change the schedule of ageing independent from resource availability. We focused on the males’ carotenoid‐based sexual signal (i.e. their red throat) and body condition as proxies of their investment in reproduction and self‐maintenance. The reproductive success of male sticklebacks depends on the expression of red coloration through improved mating success, nest defence and offspring survival (Candolin 1999a; Candolin & Tukiainen 2015). Male sticklebacks change their investment in reproduction over the season, or in response to changes in the level of male–male competition, by simultaneously adjusting the expression of red coloration and the level of courtship, territoriality and parental care (Candolin 2000; Lindström *et al*. 2009; Kim & Velando 2014). This sexual signal is costly because their maintenance requires a continual intake of carotenoids in the diet and mobilization into integuments during the breeding season (Pike *et al*. 2007). These carotenoids also have other important physiological functions related to body maintenance (Olson & Owens 1998; Blount *et al*. 2003), leading to faster somatic senescence in breeding males with a reduced carotenoid intake (Pike *et al*. 2010b). The allocation of carotenoids among these competing demands is therefore determined by a trade‐off between current reproduction and self‐maintenance, so the expression of sexual signals should ideally be considered within a life‐history context (Badyaev & Vleck 2007). Recent quantitative genetic studies have shown that individual variation in the expression of carotenoid signals has a genetic component (Evans & Sheldon 2015; Vergara, Fargallo & Martinez‐Padilla 2015). However, in species like the stickleback in which carotenoid signals change dynamically throughout the reproductive season, it may be difficult to determine this genetic contribution with only cross‐sectional measurements of the signals, hence the need for a longitudinal approach.

The aims of this study were therefore to use a longitudinal experiment to examine whether male sticklebacks change their investment in sexual signalling and consequent pattern of senescence in response to earlier environmental conditions, and if so, to examine whether this ability is genetically determined. We do the latter by exploring the quantitative genetics of temporal dynamics in sexual signal and condition (genotype‐by‐time interaction, G × T) and by testing for a genotype‐by‐environment interaction in the pattern of senescence (G × T × E). We show that animals change their senescence schedules in response to environmental conditions, and that this ability is genetically determined.

## Materials and methods

### Breeding Design and Rearing Condition

Sexually immature three‐spined sticklebacks were captured in the Rio Ulla, Galicia, Spain, in February 2013. Like in other populations near the southern edge of the species’ range (Poizat, Rosecchi & Crivelli 1999), in this population fish reproduce by spawning repeatedly throughout a single relatively long breeding season, after which they die (females produce on average 6·1 clutches between February and August; *our unpublished data*). In Iberian populations, adults disappear in summer and 2‐year‐old breeders have never been recorded (Fernández *et al*. 2015). Adult body condition declines as the breeding season progresses, and it has been suggested that harsh conditions imposed by Iberian summers are close to the species tolerance limits (Clavero, Pou‐Rovira & Zamora 2009).

Among 70 collected fish, 16 eventually mature males and 16 mature females were randomly selected and used for breeding. Breeding design and fish husbandry are fully described in a previous study (Kim & Velando 2015). Briefly, each fish bred twice with two different randomly chosen mates, producing a total of 32 full‐sib families of the F1 generation, during April–May 2013. Thus, each F1 fish had full‐sibs and maternal and paternal half‐sibs.

At age 40 days, fry in each full‐sib family were divided among two (*N* = 7 families) or four (*N* = 25 families) ‘growth tanks’ (8 L; *N *= 114 tanks), each initially housing 11 or 12 juvenile fish (*N* = 1359 individuals). Two fish per tank were subsequently removed at age 5 months to be used in another experiment (Kim 2016). The tanks were connected to four closed water systems (30 tanks per system). In each system, water was filtered for nitrification, aerated and temperature‐controlled by the combined continuous function of a mechanical filter, a circulation pump and a flow‐through water‐cooling device. Each full‐sib family was allocated to four (or two) randomly selected tank positions in the four (or two) different aquaria systems. Juvenile fish were fed to satiation daily (twice daily up to age 5 months then once a day) on a progressive diet of newly hatched *Artemia* (from hatching to age 3 months) and a commercial pelleted diet (from age 2 months onwards; Gemma Micro, Skretting, Norway). Natural photoperiod was simulated by programmed illumination.

### Temperature Manipulation

The water temperature in the growth tanks reflected the natural seasonal pattern at the sampling site of the parent fish (i.e. reaching a peak of 20 °C in August, then falling steadily) until the experimental manipulation of temperatures began in November as shown in Fig. S1. Juvenile fishes that survived until age 6 months were weighed and permanently marked with colour elastomer tags (Northwest Marine Technologies, Shaw Island, WA, USA) under a low dose of benzocaine anaesthetic to allow tracking of individual‐specific life histories (*N* = 1038 individuals). Among the four aquaria systems, two were randomly assigned to the normal winter treatment group and the other two to the warm winter group. Therefore, each family was equally allocated into the two experimental groups. Water temperature in the normal winter group was gradually reduced from 14 °C in November to 9 °C in January then increased to 14 °C in March to simulate natural temperature change in the sampling site, whereas the temperature in the warm winter group was maintained at 14 °C from November onwards throughout the winter, so that the fish experienced a more benign juvenile environment (Fig. S1, Supporting information).

### Life‐History and Nuptial Coloration

We monitored the growth tanks once or twice every week during the 2014 breeding season to record the maturation pattern of males. Between March and May, a randomly selected subsample of males that had started to produce red coloration (104 control males and 105 warm‐treated males) were allocated into individual tanks containing a sponge filter and provided with sand and polyester thread as nesting materials (for sampling details, Appendix S1, Supporting information); they were then monitored for reproductive investment across the breeding season until August.

During the 6 months of the reproductive season, each of the monitored males (*N *= 209) was shown a gravid female enclosed in a transparent glass for 5 min twice a week to prompt expression of nuptial colour and behavioural investment in nest construction and courtship (Kim & Velando 2014). Each male was repeatedly photographed and measured (standard length and body mass) every 2 weeks up to 11 times throughout the season (on average 10 times). On each occasion, the fish was placed in a small transparent water‐filled plastic box, positioned on its lateral side (either left or right to reduce handling time) using a grey sponge and photographed under standardized conditions within a black box containing LED illumination using a digital camera (Nikon D90, Nikon Corp., Tokyo, Japan) (Candolin 1999b). The photographs were scheduled so that the males were always photographed 2–3 h after the stimulation with a gravid female. By the end of August, all females in the growth tanks had stopped egg production and most males had become dull, and so we stopped presenting females and photographing males. Six of the study males died during the reproductive season (three from each treatment group).

We measured the area of red nuptial coloration (hue: 1–60 and 340–359; saturation: 50–255; intensity: 0–255) from the digital images by using image analysis software (analysis five) (Kim & Velando 2014). Relative size of the red area was calculated as a percentage of the total lateral body area. We also determined the maximum red colour area during the season in each male. Body condition of each fish at each time of measurement was calculated as the residual from a linear regression of log_10_ body mass against log_10_ standard length (*N *= 2081 repeated measurements, *r* = 0·885, *P* < 0·001) (Bolger & Connolly 1989).

In July, the level of mating competition was manipulated in order to test whether senescent males that had earlier experienced different temperature schemes differed in male–male competition strategies and terminal investment. Any effect of this competition treatment (i.e. whether presented with an attractive or dull rival) on red coloration has been taken into account in all statistical analyses (for details, Appendix S2, Supporting information).

### Statistical Analyses

We first explored the effects of the temperature manipulation on the temporal dynamics of relative red area and body condition of the male sticklebacks by using longitudinal analyses of repeated measures (for each trait, control: *N* = 1020 observations from 104 males; warm‐treatment: *N* = 1061 observations from 105 males). In a linear mixed‐effect (LME) model fitted to each trait, experimental treatment (control or warm winter), hatching date, date of initiation of breeding (i.e. first expression of red throat coloration), strength of competition (strong or weak treatment), time (unit of 2 weeks between 26 March and 21 August) and interactions of interest were included as fixed effects, and individual identity, growth tank and full‐sib family were included as nested random effects. Additionally, body condition was also analysed in a LME model fitted to body mass and including standard body length as an additional covariate (Table S1, Supporting information). Non‐significant fixed effects were dropped sequentially by using deletion tests, and then, the final LME model was fitted using the default restricted maximum likelihood (REML) to obtain estimates and significance levels of fixed effects in r (version 3.0.2).

We estimated individual and additive genetic (co)variances across time in relative red area and body condition (mass‐standard length residuals) by using pedigree‐based REML random regression animal models (RRAMs), implemented in asreml (version 3). The random regression (RR) is the most widely applied function‐valued trait approach, and when applied to longitudinal data, RR can provide a direct test of individual variation in ageing rates. We followed recently described methods to construct RRAMs for the quantitative genetics of ageing (Brommer, Rattiste & Wilson 2010; Charmantier, Brommer & Nussey 2014). Obtaining accurate estimates of genetic parameters in RRAMs requires a lot of repeated data from individuals from a well‐connected pedigree (Wilson *et al*. 2010). The estimation of the additive genetic (co)variances in this study was based on 2081 repeated measures and full‐ and half‐brother relationships of 209 F1 individuals with very high relatedness (pedantics, Morrissey & Wilson 2010; pairwise relatedness (≥0·25) of the pedigree = 0·086; average parental sibship size = 13). Simple animal model analyses without a RR function showed that a significant and high proportion of the total phenotypic variance was explained by additive genetic effects (*h* ^2^, heritability) for relative red area and body condition, while permanent environment (*pe* ^2^, individual‐specific), maternal (*m* ^2^, clutch‐specific) and common environment (*c* ^2^, growth tank‐specific) effects were negligible (Table S2, Supporting information). Therefore, clutch and growth tank were not included as random effects in the RRAM analyses, but individual identity was included to account for the repeated sampling and individual (co)variance. Body condition was analysed using only mass‐standard length residuals and not body mass, because the latter requires including standard length as a covariate. Since body length itself is a heritable trait that is genetically correlated with body mass, including this covariate as a fixed effect in the RRAM analysis can severely bias estimates of the genetic parameters (Wilson 2008).

In the RRAMs, relative red area and body condition in individual *i* was expressed as a function of standardized time *t* (unit of 2 weeks) to a scale from −1 to 1. By using univariate RRAMs fitted to the control and warm winter groups separately, we first estimated the between‐individual variations in average trait (elevation) and ageing rates (linear and quadratic slopes of the trait measurement over standardized time) and the covariances between elevation and slopes. Then, the individual level RR function (*I* × *T*, where *T* = time) was partitioned further to estimate the contributions of genetic effects (*G*) and non‐genetic effects due to individual‐specific past environmental conditions (permanent environment effects, PE) to the individual variations in average trait and ageing rate (*G* × *T* and PE × *T*). Thus, we fitted the hierarchical univariate models for a trait *T* of individual *i* at time *t* as:

$$T_{i,t}=\mu +timeF+compF+f\left(pe_{xi},t\right)+f\left(a_{xi},t\right)+\varepsilon_{i,t}.$$

We fitted the models in hierarchical order, starting from only fixed effects and heterogeneous residual error (ɛ_*i,t*_). Apart from the overall trait mean μ, *timeF* and *compF* are factorial fixed effects that denote time (an 11 level factor) and competition treatment (a two level factor). Then, *f(ind*_0*i*_ *,t)* was fitted as the RR function of zero order (*x* = 0), which specifies the between‐individual variation in the average trait. When *x* = 0, the variance across individuals (*ind*_0_) is estimated at the average time value of the individual‐specific variation. In subsequent models, *f(ind*_0*i*_ *,t)* was partitioned into additive genetic and permanent environment components, *f(a*_0*i*_ *,t)* and *f(pe*_0*i*_ *,t)*, then different orthogonal polynomial functions of time were specified to examine how the effects differed between constant (zero order, *pe*_0_ and *a*_0_), linear (first order, *pe*_1_ and *a*_1_) and quadratic forms (second order, *pe*_2_ and *a*_2_). Therefore, *a*_0_ and *pe*_0_ refer to the variances in reaction norm elevations, representing additive genetic and permanent environmental effects on the average trait value; *a*_1_, *a*_2_, *pe*_1_ and *pe*_2_ refer to the variances in reaction norm slopes, representing genetic and permanent environmental effects on ageing rates of either linear or quadratic form. In addition, the RRAMs also estimated the covariances among elevation and slopes. Significance of (co)variance components was assessed by using model comparisons based on likelihood ratio tests (LRT).

Senescence (i.e. a decline) in red coloration commenced in mid‐May (see Results). Therefore, in order to explicitly test for *G* × *T* and PE × *T* effects, we also explored ageing patterns from the onset of senescence by fitting RRAMs to a subset of the red coloration data (i.e. measurements from 21 May–21 August only). The univariate model structures were the same as above, but here we considered linear reaction norms only by fitting polynomial functions of a zero and first order because the analyses based on the full data set suggested linear reaction norm patterns during this period.

The univariate RRAMs that examined rates of senescence of sexual coloration and body condition considered the two temperature treatment groups separately. To explicitly test whether the patterns of ageing and their genetic and permanent environment basis (i.e. *G* × *T* and PE × *T*) differed between the control and warm winter groups, we used bivariate RRAMs. A *G* × *T* × *E* interaction is expected if genetic effects on ageing differ between environments. We combined the data sets and pedigree information from the two groups and tested for *G* × *T* × *E* and PE × *T* × *E* interactions. For example, relative red area of the two treatment groups was fitted as two response variables to a bivariate RRAM. Here, all covariances between the two treatment groups were constrained to be zero by modelling the genetic, permanent environment and residual variances as treatment‐specific matrices. For the comparison between the treatment groups, the respective (co)variance components (i.e. elevation, slope and covariance between elevation and slope) in the two groups were constrained to be equal, assuming that they have the same genetic and permanent environmental variances in average trait and ageing rate. The likelihood optimized under the constrained model was compared to that of the unconstrained model by using LRT (Husby *et al*. 2010).

## Results

### Effects of Overwinter Temperature on Seasonal Dynamics of Colour and Condition

**Figure 1:**
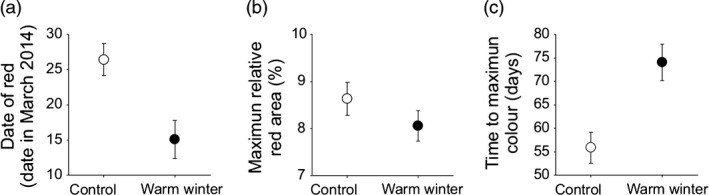
Comparisons of male traits between the control and warm winter groups. Mean ± SE (a) date at which red ornamentation was first detected, (b) maximum value over the season for relative area of red coloration and (c) time taken to reach maximum red coloration, calculated as the time from when red coloration was first detected.

The date of initiation of breeding was significantly earlier for the warm‐treated than the control males (LME: treatment: *t*_80_ = −3·664, *P* < 0·001, hatching date: NS; Fig. 1a). However, while the maximum size of a male's red area (relative to his body size) did not differ significantly between the two treatment groups (*t*_80_ = −1·159, *P* = 0·250; Fig. 1b), the warm‐treated males took longer from first signs of red to reaching their maximum coloration than did the controls, despite having started to become red earlier (treatment: *t*_80_ = 3·623, *P* < 0·001; Fig. 1c). Males born earlier were quicker to reach their peak red coloration (hatching date: *t*_30_ = 3·086, *P* = 0·004), and also had a higher peak coloration (*t*_30_ = −2·194, *P *= 0·036).

**Figure 2:**
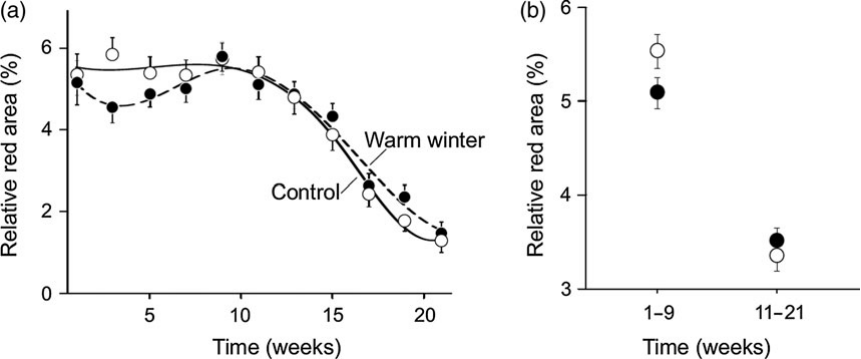
Temporal change in relative red area with respect to the winter temperature treatment. (a) Changes in mean ± SE relative red area over the reproductive season from 26 March 2014 to 21 August 2014. For illustrative purpose, adjusted splines are also presented. (b) The average values before and after the onset of senescence (1st–9th week and 11th–21st week, respectively).

Males from both temperature treatment groups showed a decline in coloration from the ninth week onwards (Fig. 2a), but this decline was faster in control males, as indicated by a significant treatment × time interaction (Table 1). In an additional analysis, we tested whether the investment in early and late coloration differed between the two temperature treatment groups by comparing the colour measurements between 1st–9th week and 11th–21st week. The control males made a proportionally greater investment in coloration during the early season compared to the warm‐treated males (significant treatment × factorial time effect: *t*_1869_ **= 2·038, *P* = 0·042; see Fig. 2b).

**Table 1:** Results from the minimum adequate linear mixed models of relative size of red nuptial colour area (percentage of the total lateral body area) and body condition (mass‐standard length residuals) of male sticklebacks. Random effects: fish identity nested within growth tank and family

Fixed effects	Relative red area				Body condition			
	Estimate ± SE	d.f.	*t*	*P*	Estimate ± SE	d.f.	*t*	*P*
Intercept	14·763 ± 2·109	1868	7·001	<0·001	5·262 ± 1·149	1866	4·579	<0·001
Treatment (warm)	−1·040 ± 0·355	80	−2·932	0·004	−0·622 ± 1·390	80	−0·447	0·656
Time	0·370 ± 0·045	1868	8·274	<0·001	−0·503 ± 0·037	1866	−13·508	<0·001
Time^2^	−0·025 ± 0·002	1868	−13·754	<0·001	0·005 ± 0·000	1866	13·058	<0·001
Hatching date	–			NS^b^	−			NS
Competition (strong)	−0·874 ± 0·221	1868	−3·959	<0·001	−			NS
Date of red (DR)^a^	−0·029 ± 0·006	95	−5·030	<0·001	−0·010 ± 0·003	94	−3·253	0·002
Treatment × time	0·059 ± 0·020	1868	2·885	0·004	0·201 ± 0·047	1866	4·233	<0·001
Treatment × time^2^	–			NS	−0·001 ± 0·001	1866	−2·229	0·026
Treatment × DR	–				0·001 ± 0·003	94	0·263	0·793
Time × DR	–				0·001 ± 0·000	1866	7·618	<0·001
Treatment × time × DR	–			NS	−0·000 ± 0·000	1866	−3·292	0·001

*N* = 2081 observations, *N* = 209 individuals.

a Date at which red ornamentation was first detected.

b NS, non‐significant.

Body condition also declined over time at a faster rate in the control males than in the warm‐treated males resulting in a significant temperature × time and temperature × time^2^ interactions (Table 1; Fig. S2, Supporting information). There was also a significant temperature × date of red × time interaction, with a faster decline in body condition in those control males that had become red earliest in the season, but no such effect in warm‐treated males (Table 1; Fig. S2, Supporting information). The analysis of body mass including standard length as a covariate yielded similar results to the analysis of body condition calculated as mass‐standard length residuals (Table S1, Supporting information).

### Quantitative Genetics of Ageing

In the analysis of the full data set, the relative area of the red throat showed significant linear and quadratic individual‐by‐time interactions [PE × *T* (*pe*_1_) and PE × *T* ^2^ (*pe*_2_)] in both control and warm winter groups (*P *< 0·001; Table S3, Supporting information). There was significant improvement in the model when fitting a *G* × *T* (*a*_1_) component (control: *P* = 0·015; warm: *P* = 0·036); the inclusion of a second‐order *a*_2_ term did not significantly improve the model (Table S3, Supporting information). In the analysis of changes in relative red area from the onset of senescence onwards (≥9th week), there was significant individual (PE × *T*) and genetic (*G* × *T*) variance in the rate of senescence in the control males, but only the individual variance (PE × *T*) was significant in the warm‐treated males (Table 2). The bivariate RRAM analysis demonstrated that the individual and genetic variance in the rate of senescence differed significantly between the control and warm‐treated males, suggesting genotype‐by‐environment and permanent environment‐by‐environment interactions for the rate of senescence (PE × *T* × *E*: χ^2^_3_ = 8·84, *P* = 0·031; *G* × *T* × *E*: χ^2^_3_ = 22·22, *P* < 0·001).

**Table 2:** Results from the univariate random regression animal model analyses of relative red area from the onset of senescence (9th week onward) in the control and warm winter treatment groups. *V*_I_ is the between‐individual variance in relative red area and *V*_A_ is the additive genetic variance. PE × T and G × T denote the permanent environment and additive genetic variances in the rate of senescence. The significance of each variance component was tested by comparing between different hierarchical models (a) based on a likelihood ratio test. For example, *V*_I_ was tested based on the comparison between model 1 and model 2. The REML estimated variances and covariances between elevation and slope of the best‐fit models are given with their SEs in brackets

Model selection								
Model^a^	Tested component	d.f.	Control			Warm winter		
			LogL	χ^2^	*P*	LogL	χ^2^	*P*
1	–		−1181·88			−1196·11		
2	*V*_I_ (*ind*_0_)	1	−1077·55	208·66	<0·001	−1055·04	282·14	<0·001
3	*V*_A_ (*a*_0_)	1	−1074·27	6·56	0·010	−1052·47	5·14	0·023
4	PE × T (*pe*_1_)	2	−1024·69	99·16	<0·001	−985·22	134·50	<0·001
5	G × T (*a*_1_)	2	−1019·43	10·52	0·005	−984·16	2·128	0·345

RRAM (co)variances of the best‐fit models				
Variance–covariance	Control (model 5)		Warm winter (model 4)	
	Parameter estimate	*P*	Parameter estimate	*P*
*V*ɛ_−1_	9·266 (1·714)		5·068 (1·068)	
*V*ɛ_−0·667_	4·408 (0·907)		4·370 (0·803)	
*V* ɛ_−0·333_	4·995 (0·853)		3·223 (0·561)	
*V* ɛ_0_	6·892 (1·063)		3·544 (0·562)	
*V* ɛ_0·333_	2·987 (0·495)		2·405 (0·404)	
*V* ɛ_0·667_	1·029 (0·341)		1·220 (0·351)	
*V* ɛ_1_	3·423 (0·787)		3·210 (0·745)	
*V pe*_0_	1·924 (1·293)		3·339 (1·173)	
*Cov pe*_0*,*_ *pe*_1_	0·651 (1·030)	0·527	−0·863 (0·597)	0·183
*V pe*_1_	1·648 (1·373)		4·941 (0·862)	
*V a*_0_	3·818 (1·891)		2·337 (1·422)	
*Cov a*_0*,*_ *a*_1_	−2·685 (1·532)	0·026	–	
*V a*_1_	3·656 (1·921)		–	

a Model 1: *T*_*i,t*_ = μ + *timeF* + *compF* + ɛ_*i,t*._

Model 2: *T*_*i,t*_ = μ + *timeF* + *compF* + *f(ind*_0*i*_ *,t)* + ɛ_*i,t*._

Model 3: *T*_*i,t*_ = μ + *timeF* + *compF* + *f(pe*_0*i*_ *,t)* + *f(a*_0*i*_ *,t)* + ɛ_*i,t*._

Model 4: *T*_*i,t*_ **= μ + *timeF* + *compF* + *f(pe*_1*i*_ *,t)*  + *f(a*_0*i*_ *,t)* + ɛ_*i,t*_

Model 5: *T*_*i,t*_ = μ *+ timeF* + *compF* + *f(pe*_1*i*_ *,t) + f(a*_1*i*_ *,t) + *ɛ_*i,t*_

**Figure 3:**
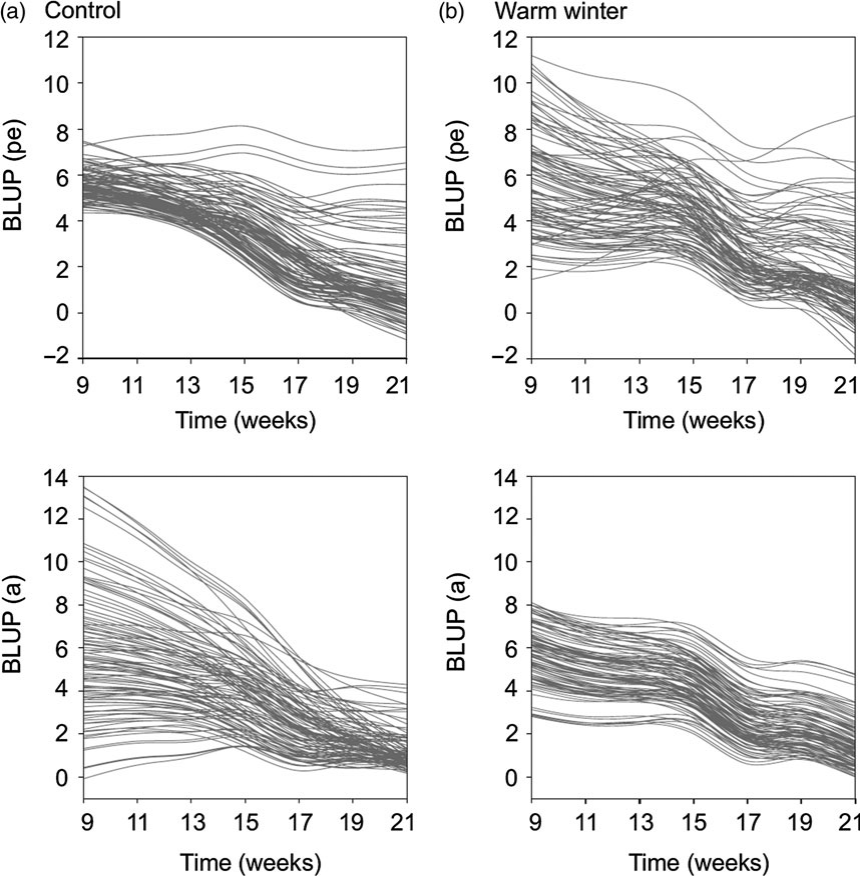
Reaction norm plots of the trajectories for red coloration over time since the onset of senescence (9th week onwards) of individual male sticklebacks from the (a) control and (b) warm winter temperature treatments (control: *N* = 104 individuals; warm winter: *N* = 105). Each line represents the predicted trajectory of a single individual either at the permanent environment (*pe*) or additive genetic (*a*) level. Trajectories are based on the fixed effects (average trait and ageing rate) and individual‐specific elevation and slope from the best linear unbiased predictor (BLUP) values of model 5 in Table 2. BLUPs are used here to merely illustrate the PE × T × E and G × T × E patterns, but were not used in the statistical analyses.

Ageing trajectories of individual males were visualized based on the best linear unbiased predictor (BLUP) values of the best‐fit univariate RRAMs, in order to illustrate the patterns of the significant PE × *T* × *E* and *G* × *T* × *E* interactions (Fig. 3). The permanent environment variance for red coloration increased over time in the control males but remained at the same level in the warm‐treated males. Most importantly, the additive genetic variance for red coloration was large at the onset of senescence then decreased over time in the control but not the warm‐treated males (Fig. 3). In other words, the differences among genotypes converged over time in the control males. In these males, the average level of red coloration since the onset of senescence (i.e. the elevation) and the senescence rate (the linear slope) were negatively correlated at the additive genetic level (elevation–slope correlation *r*_a0,a1_ = −0·719 ± 0·221), and *Cov*_a0,a1_ differed significantly from zero (Table 2). Thus, in the control group only, genotypes expressing larger average areas of sexual ornamentation during this period of the breeding season showed a faster senescence compared to those with weaker coloration (Fig. 3). In contrast, genotypes of warm‐treated males showed similar ageing patterns (Fig. 3).

Univariate RRAMs of body condition suggested the presence of a significant *I *× *T* ^2^ interaction in both control and warm winter groups (*P* < 0·001; Table S3, Supporting information), with no difference in pattern between them (bivariate RRAM: χ^2^_6_ = 5·37, *P* = 0·497). In the control males (but not in the warm‐treated males), this could be decomposed into significant *G* × *T* ^2^ and PE × *T* ^2^ components (*P* = 0·016), since additive genetic variance for body condition increased over the breeding season whereas permanent environment variance decreased over the same period (Fig. S3, Supporting information). There was no significant covariance between any elevation–slope combinations of body condition in either temperature treatment group (Table S3, Supporting information).

## Discussion

The association shown here between the effort invested by male sticklebacks in sexual signalling early in the breeding season and the rate of ageing later in the season provides experimental support for the disposable soma theory. Meanwhile, our results on the quantitative genetics of senescence in sexual signals support the antagonistic pleiotropy theory of ageing. But perhaps most importantly, we experimentally demonstrated the expression of significant G × E interactions influencing the pattern of senescence. The environment experienced by males prior to the breeding season influenced their investment in early reproduction and subsequent rate of ageing. The difference in *G* × *T* (genetic variance in the rate of senescence) between the experimental groups suggests that the importance of antagonistic pleiotropic effects depends on environmental conditions, such that these can shape senescence schedules in natural populations through heritable differences in life‐history plasticity.

In accordance with previous studies of sticklebacks showing that warm temperatures prior to the breeding season prompt a slower lifestyle and delayed senescence (Lee, Monaghan & Metcalfe 2012, 2013), the warm‐treated males in our study reduced their early investment in sexual signals (as evidenced by the delayed peak of coloration), then exhibited a slower rate of senescence in both sexual signal and body condition compared to the controls. This may be a programmed response to optimize the temporal pattern of investment in sexual signalling according to a male's life expectancy; an optimization model of this scenario, developed within a game‐theory framework, predicted that males in better nutritional condition should show restraint in signalling early in the season whereas those in poorer condition should signal at their maximal rate, even if that causes a more rapid decline (Lindström *et al*. 2009). Our results fit these predictions, since the control males invested more in sexual signalling during the early season than did those that had experienced the benefits of a warm winter, but then showed a more rapid decline in both coloration and body condition as the season progressed.

Moreover, only among the control males was there a significant relationship between the extent of early investment in sexual ornamentation and the rate at which body condition declined, suggesting that the trade‐off was more acute in the males experiencing the harsher pre‐breeding environment. Food was not limiting at any time in either temperature treatment group, so differences in macronutrient levels are unlikely to have been the cause of the more rapid deterioration in ornamentation and body condition of the control males. Instead, the effect may be driven by a greater early investment of carotenoids into sexual signalling. Carotenoids can be limiting in the diet, and breeders should continuously invest this limiting resource if they are to maintain red coloration during the season (Pike *et al*. 2007). In addition to their role in creating the red coloration of the male stickleback, carotenoids have important physiological functions as antioxidants. The early investment of this resource into colourful ornaments can, thus, constrain physiological condition through reduced antioxidant capacity (Metcalfe & Alonso‐Alvarez 2010). Indeed, it has already been shown that male sticklebacks that invest a disproportionate fraction of their carotenoids into sexual signals tend to senesce faster and die sooner (Pike *et al*. 2007, 2010a,b). Therefore, our temperature manipulation likely altered the pattern of ageing by influencing the trade‐off between sexual signalling and somatic maintenance, supporting the disposable soma theory of ageing at the phenotypic level.

Two of the important findings of the present study are that variation in nuptial coloration and body condition has high and significant heritability (overall *h* ^2^ > 0·8, after controlling for maternal and environmental effects), and that the pattern of ageing also showed heritable variation, at least in control males. While the three‐spined stickleback has frequently been used as a model system to study the evolution of sexual signals (e.g. Boughman, Rundle & Schluter 2005), there has been little evidence of the heritability of its red ornamentation (but see Bakker 1993). The strong genetic variance in the red ornament evident from our study indicates the evolutionary potential of this trait through female choice (Griffith, Parker & Olson 2006). Furthermore, the genetic variance in the temporal dynamics of both the sexual signal and body condition (i.e. significant *G* × *T* effects) suggests that a male's strategy for the timing of allocation of carotenoids and other resources into sexual signalling vs. somatic maintenance through the reproductive season is heritable and should also be subject to evolution under selection. An interesting question here is how the genetic variation in senescence rates is maintained – possibly due to fluctuating selection (Robinson *et al*. 2008) or alternative reproductive strategies (Sinervo & Lively 1996). While it is often thought that males are selected to pursue a ‘live fast, die young’ strategy, involving high rates of somatic damage and rapid ageing in comparison with females, current evidence offers little support for this (Bonduriansky *et al*. 2008). Interestingly, a study on the three‐spined stickleback showed that female preference for redder males becomes stronger towards the end of the season (Lindström *et al*. 2009), suggesting that temporally dynamic selection forces may promote different male reproductive strategies.

Most interestingly, this present study provides evidence for genotype‐by‐environment interactions in the rate of senescence (*G* × *T* × *E* in red coloration and body condition), which support two important ideas. First, the data support the antagonistic pleiotropy theory of ageing that predicts a genetic link between enhanced performance earlier in life and an accelerated rate of senescence (Kirkwood & Rose 1991). While two previous quantitative genetic studies based on long‐term population data of a bird and a mammal have explored antagonistic pleiotropy by focussing on the demonstration of a negative genetic correlation between early and late reproductive performance (Charmantier *et al*. 2006; Nussey *et al*. 2008), our experiment tested the theory by effectively suppressing the expression of pleiotropic genes in one treatment group. Thus, the trade‐off between early and late benefits, recently highlighted in a review of studies of ageing in wild populations (Lemaître *et al*. 2015), was evident in the control group (with genotypes that were redder in the early breeding season showing a faster senescence later in the season), but was removed when we experimentally induced a reduction in the investment in sexual signalling in the early part of the breeding season (through the warm winter treatment). Second, this study provides evidence that the extent to which genetic differences influence ageing varies across environments. The phenotypic differences in investment in sexual signalling and ageing between the two experimental groups were due to the flexible responses of the same genotypes to the environmental conditions. Mechanisms such as differential gene expression or epigenetic modulation across environments may underlie *G* × *T* × *E* effects (Schlichting & Pigliucci 1998). In particular, the *G* × *T* × *E* in red coloration appeared mainly because the redder genotypes reduced their colour expression early in the breeding season after experiencing benign environmental conditions. Although the temperature at the beginning of the breeding season was the same in the two environmental treatments, the males raised in warm winter conditions experienced lower seasonality than the control males. It is possible that the reduced seasonal difference in temperatures might prompt the warm‐treated breeders, particularly those of redder genotypes, to suppress the expression of red ornamentation via a downregulation of their sexual hormones (Gonzalez *et al*. 2001).

This study provides interesting insights into the role of environment in the evolution of life histories and sexual signals. We have shown experimentally that environmental conditions experienced by individuals during early life can determine life‐history integration and trade‐offs by shaping their investment in sexual signalling and senescence schedules. Importantly, genetic variation in the rate of senescence was reduced to a non‐significant level among individuals raised in a benign environment. Therefore, more benign environmental conditions weaken the scope for evolution in the pattern of senescence. The environmental dependence may also explain the maintenance of genetic variation in life‐history and secondary sexual traits against the eroding effects of strong natural and sexual selection in natural populations.

## Data accessibility

Data available from the Dryad Digital Repository http://dx.doi.org/10.5061/dryad.s6h6b (Kim, Metcalfe & Velando 2015).

## Supporting information


**Appendix S1.** Sampling of monitored sticklebacks.
**Appendix S2.** Male–male competition experiment.
**Table S1.** Results from the linear mixed model of body mass of male sticklebacks.
**Table S2.** Variance components, heritability and environmental effects from univariate animal models.
**Table S3.** Results from the random regression animal models of relative red area and body condition.
**Figure S1.** Seasonal change in the water temperature of the F1 growth tanks of normal and warm winter treatment groups.
**Figure S2.** Relationship between seasonal change in body condition and time of maturation of males from the control and warm winter groups.
**Figure S3.** Plots of the reaction norms for body condition of individual sticklebacks of the control and warm winter schemes.
