# Supplementary material for: A benign juvenile environment reduces the strength of antagonistic pleiotropy and genetic variation in the rate of senescence
Source: J Anim Ecol. 2015 Dec 19;85(3):705–14. doi: 10.1111/1365-2656.12468 (PMC4991295; doi:10.1111/1365-2656.12468)
Supplement: Supplementary file 1 — Appendix S1. Sampling of monitored sticklebacks. Appendix S2. Male–male competition experiment. Table S1. Results from the linear mixed model of body mass of male sticklebacks. Table S2. Variance components, heritability and environmental effects from univariate animal models. Table S3. Results from the random regression animal models of relative red area and body condition. Figure S1. Seasonal change in the water temperature of the F1 growth tanks of normal and warm winter treatment groups. Figure S2. Relationship between seasonal change in body condition and time of maturation of males from the control and warm winter groups. Figure S3. Plots of the reaction norms for body condition of individual sticklebacks of the control and warm winter schemes. [file JANE-85-705-s001.pdf]

Supporting information

Appendix S1. Sampling of monitored sticklebacks

It should be noted that the F1 fish were also used in other studies, so that only a fraction of the experimental population was used in the present study. We monitored all the growth tanks once or twice every week from January 2014 to record the maturation pattern of males and females. A total of 327 females spawned repeatedly during the breeding season. The females were maintained in the growth tanks and presented to the males when gravid.

A total of 189 males from the control and 203 males from the warm winter group started to express red nuptial colour on their throat between February and May 2014. Males that experienced the warm winter temperature treatment first started to express red throat colour earlier in the season than the control males (on average, 5 April in the control and 23 March in the warm winter group; $t_{390} = 4.348$, $P < 0.001$). Between March and May randomly selected males that had started to produce red colouration (104 control males and 105 warm-treated males) were allocated into individual tanks; they were then monitored for reproductive investment across the breeding season until August. Those that started to turn red before March were kept in their growth tanks until temperature of the two treatment groups was the same again (Fig. S1). Although we tried to select subsamples that represented all mature males, the randomly selected samples was slightly biased toward those maturing earlier in both treatment groups because we were short of individual tanks later the season (see results, Fig. 1a). At the end of the reproductive season, the males were returned to their original growth tanks for tracking of post-reproductive lifespan (to be reported in a future publication).

Appendix S2. Male-male competition experiment

In July the level of mating competition was manipulated in order to test whether males that had earlier experienced different temperature schemes differ in male-male competition strategies and terminal investment. We randomly assigned one half of the males in each treatment group to the strong competition regime and the rest to the weak competition regime. Males of the strong competition group were challenged with a dummy stickleback with red colour on throat and belly, simulating an attractive and competitive rival, while males of the weak competition were presented with a dull coloured dummy with a yellow spot on its throat (for details, see Kim & Velando 2014). Territorial invasion was simulated by moving the dummy within 5 cm of each focal fish without physical contact during 5 min × twice a week × four weeks.

Manipulation of the level of mating competition had a significant effect on the relative extent of sexual ornamentation, since after a month of treatment the males challenged with a red-throated dummy fish had a smaller red area than those challenged with a dull dummy, but this effect did not differ between the control and warm-treated males (LME: competition treatment: $t_{91} = -3.245$, $P = 0.002$; temperature treatment: NS; competition × temperature treatment: NS).

Since the optimal signalling level of a male depends on both his intrinsic state and the signalling effort of other males in the population (Lindström *et al.* 2009; Kim & Velando 2014), we expected that males that had deteriorated most (here, the control males of the temperature manipulation experiment) will increase their terminal investment in sexual signals when the level of mating competition is strong. However, our results from the competition experiment showed no evidence for this, probably because the males had already deteriorated to beyond the critical level by the time of the competition treatment (McNamara *et al.* 2009). The results of the analyses of body condition suggest that physiological condition may constrain senescent males from investing overly in sexual signals.

References

Kim, S.-Y. & Velando, A. (2014) Stickleback Males Increase Red Coloration and Courtship Behaviours in the Presence of a Competitive Rival. *Ethology,* **120,** 502-510.

Lindström, J., Pike, T.W., Blount, J.D. & Metcalfe, N.B. (2009) Optimization of resource allocation can explain the temporal dynamics and honesty of sexual signals. *American Naturalist,* **174,** 515-525.

McNamara, J.M., Houston, A.I., Barta, Z., Scheuerlein, A. & Fromhage, L. (2009) Deterioration, death and the evolution of reproductive restraint in late life. *Proceedings of the Royal Society of London B: Biological Sciences,* **276,** 4061-4066.

Table S1. Results from the minimum adequate linear mixed models of body mass of male sticklebacks. Random effects: fish identity nested within growth tank and family. N = 2081 observations, N = 209 individuals

Fixed effects	Estimate ± SE[a]	d.f.	t	P
Intercept	-8.978 ± 1.224	1865	-7.335	< 0.001
Standard length	0.549 ± 0.015	1865	37.734	< 0.001
Temperature (warm)	-1.019 ± 0.982	80	-1.038	0.303
Time	-0.474 ± 0.027	1865	-17.558	< 0.001
Time^2	0.004 ± 0.000	1865	12.205	< 0.001
Hatching date	-			NS[c]
Competition (strong)	-			NS
Date of red (DR)[b]	-0.009 ± 0.002	94	-3.783	< 0.001
Treatment × time	0.205 ± 0.034	1865	5.955	< 0.001
Treatment × time^2	-0.001 ± 0.000	1865	-2.374	0.018
Treatment × DR	0.002 ± 0.003	94	0.826	0.411
Time × DR	0.001 ± 0.000	1865	11.876	< 0.001
Treatment × time × DR	-0.000 ± 0.000	1865	-4.837	< 0.001

[a]Estimates and their SE × 10^{-1}.

[b]Date at which red ornamentation was first detected.

[c]NS: Nonsignificant.

Table S2. Variance components (V_A, additive genetic variance; V_{PE}, individual-specific permanent environment variance; V_M, clutch-specific maternal variance; V_C, growth tank-specific common environment variance; and V_P, total phenotypic variance), heritability (h^2), environmental effects (pe^2, m^2 and c^2) and their SE from univariate animal models. *P*-values are given in brackets

	Relative red area		Body condition	
	Control	Warm winter	Control	Warm winter
V_A	4.390 ± 2.552	2.890 ± 1.800	0.371 ± 0.192	0.107 ± 0.188
	($P < 0.001$)	($P < 0.001$)	($P < 0.001$)	($P = 0.063$)
V_{PE}	0.644 ± 1.407	1.262 ± 1.060	0.076 ± 0.106	0.421 ± 0.123
	($P = 0.671$)	($P = 0.299$)	($P = 0.502$)	($P = 0.007$)
V_M	0.092 ± 0.802	0.251 ± 0.671	0.008 ± 0.058	0.044 ± 0.096
	($P = 0.888$)	($P = 0.655$)	($P = 0.882$)	($P = 0.626$)
V_C	0	0	0	0
	($P = 1$)	($P = 1$)	($P = 1$)	($P = 1$)
V_P	5.127 ± 1.068	4.403 ± 0.843	0.455 ± 0.086	0.572 ± 0.085
h^2	0.856 ± 0.388	0.656 ± 0.345	0.815 ± 0.323	0.187 ± 0.324
pe^2	0.126 ± 0.288	0.287 ± 0.262	0.167 ± 0.252	0.737 ± 0.216
m^2	0.018 ± 0.157	0.057 ± 0.152	0.018 ± 0.127	0.077 ± 0.168
c^2	0	0	0	0

Table S3. Results from the random regression animal models of relative red area and body condition of male sticklebacks in the control and warm winter treatment groups. Reported statistics are from comparison with the higher ranking model and the *P*-value for the associated LRT. The REML estimated variances of the best fit models are given with their SE in brackets

(a) Relative red area								
Model [a]	Tested component	d.f.	Control			Warm winter		
			LogL	χ^2	*P*	LogL	χ^2	*P*
1	-		-1791.46			-1806.47		
2	V_I (ind_0)	1	-1656.86	269.20	< 0.001	-1653.59	305.76	< 0.001
3	V_A (a_0)	1	-1650.26	13.20	< 0.001	-1648.05	11.08	< 0.001
4	PE×T (pe_1)	2	-1605.72	89.08	< 0.001	-1532.00	232.10	< 0.001
5	PE×T^2 (pe_2)	3	-1557.55	96.34	< 0.001	-1494.07	75.86	< 0.001
6	G×T (a_1)	2	-1553.34	8.42	0.015	-1490.74	6.66	0.036
7	G×T^2 (a_2)	3	-1551.29	4.10	0.251	-1489.99	1.50	0.682

RRAM (co)variances of the best fit models				
Variance-covariance	Control (model 6)		Warm winter (model 6)	
	Parameter estimate	*P*	Parameter estimate	*P*
$V\varepsilon_{-1}$	14.822 (3.854)		8.020 (1.958)	
$V\varepsilon_{-0.8}$	7.385 (1.634)		5.232 (1.054)	
$V\varepsilon_{-0.6}$	6.0161 (1.118)		3.171 (0.623)	
$V\varepsilon_{-0.4}$	5.3903 (0.920)		5.275 (0.857)	
$V\varepsilon_{-0.2}$	9.270 (1.445)		5.214 (0.836)	
$V\varepsilon_{0}$	5.163 (0.888)		4.764 (0.774)	
$V\varepsilon_{0.2}$	3.951 (0.724)		3.276 (0.569)	
$V\varepsilon_{0.4}$	5.556 (0.911)		3.372 (0.562)	
$V\varepsilon_{0.6}$	3.023 (0.525)		2.677 (0.458)	
$V\varepsilon_{0.8}$	1.581 (0.379)		1.791 (0.385)	
$V\varepsilon_{1}$	1.983 (0.714)		1.932 (0.608)	
$V\,pe_0$	1.576 (1.128)		1.102 (0.996)	
$Cov\,pe_0, pe_1$	1.212 (0.851)	0.151	0.538 (0.783)	0.462
$V\,pe_1$	2.212 (1.141)		3.716 (1.218)	
$Cov\,pe_0, pe_2$	-2.227 (0.604)	< 0.001	-2.052 (0.436)	< 0.001
$Cov\,pe_1, pe_2$	-1.111 (0.692)	0.128	-0.085 (0.497)	0.841
$V\,pe_2$	4.876 (0.996)		2.461 (0.563)	

$V\, a_0$			3.473 (1.621)			3.959 (1.589)		
$Cov\, a_0, a_1$			-2.085 (1.192)		0.027	-1.551 (1.115)		0.107
$V\, a_1$			2.183 (1.317)			1.848 (1.271)		
(b) Body condition								
Model [a]	Tested component	d.f.	Control			Warm winter		
			LogL	χ^2	*P*	LogL	χ^2	*P*
1	-		-303.03			-372.32		
2	V_I (ind_0)	1	-184.83	236.41	< 0.001	263.38	1271.39	< 0.001
3	V_A (a_0)	1	-195.61	21.56	< 0.001	265.09	3.414	0.065
4	PE×T (pe_1)	2	300.25	991.71	< 0.001	353.25	176.32	< 0.001
5	PE×T^2 (pe_2)	3	333.54	66.59	< 0.001	392.27	78.03	< 0.001
6	G×T (a_1)	2	334.811	2.54	0.281	-		
7	G×T^2 (a_2)	3	339.996	10.37	0.016	-		
RRAM (co)variances of the best fit models								
Variance-covariance			Control (model 7)			Warm winter (model 5)		
			Parameter estimate		*P*	Parameter estimate		*P*
$V\varepsilon_{-1}$			0.090 (0.036)			0.161 (0.043)		
$V\varepsilon_{-0.8}$			0.094 (0.023)			0.073 (0.018)		
$V\, \varepsilon_{-0.6}$			0.211 (0.035)			0.059 (0.012)		
$V\, \varepsilon_{-0.4}$			0.068 (0.013)			0.089 (0.015)		
$V\, \varepsilon_{-0.2}$			0.061 (0.011)			0.093 (0.015)		
$V\, \varepsilon_0$			0.072 (0.013)			0.084 (0.014)		
$V\, \varepsilon_{0.2}$			0.080 (0.014)			0.078 (0.013)		
$V\, \varepsilon_{0.4}$			0.103 (0.017)			0.071 (0.012)		
$V\, \varepsilon_{0.6}$			0.070 (0.013)			0.073 (0.011)		
$V\, \varepsilon_{0.8}$			0.091 (0.019)			0.079 (0.015)		
$V\, \varepsilon_1$			0.121 (0.033)			0.051 (0.021)		
$V\, pe_0$			0.087 (0.097)			0.573 (0.081)		
$Cov\, pe_0, pe_1$			-0.035 (0.045)		0.419	0.022 (0.032)		0.484
$V\, pe_1$			0.173 (0.046)			0.147 (0.024)		
$Cov\, pe_0, pe_2$			-0.014 (0.039)		0.723	-0.041 (0.026)		0.124
$Cov\, pe_1, pe_2$			-0.018 (0.025)		0.479	-0.014 (0.015)		0.334
$V\, pe_2$			0.008 (0.030)			0.084 (0.017)		
$V\, a_0$			0.371 (0.156)					
$Cov\, a_0, a_1$			0.067 (0.060)		0.239			

$V\ a_1$	0.036 (0.042)	
$Cov\ a_{0,}\ a_2$	-0.050 (0.060)	0.411
$Cov\ a_{1,}\ a_2$	-0.000 (0.031)	1
$V\ a_2$	0.087 (0.045)	

[a] Model 1: $T_{i,t} = \mu + timeF + compF + \varepsilon_{i,t}$

Model 2: $T_{i,t} = \mu + timeF + compF + f(ind_{0i},t) + \varepsilon_{i,t}$

Model 3: $T_{i,t} = \mu + timeF + compF + f(pe_{0i},t) + f(a_{0i},t) + \varepsilon_{i,t}$

Model 4: $T_{i,t} = \mu + timeF + compF + f(pe_{1i},t) + f(a_{0i},t) + \varepsilon_{i,t}$

Model 5: $T_{i,t} = \mu + timeF + compF + f(pe_{2i},t) + f(a_{0i},t) + \varepsilon_{i,t}$

Model 6: $T_{i,t} = \mu + timeF + compF + f(pe_{2i},t) + f(a_{1i},t) + \varepsilon_{i,t}$

Model 7: $T_{i,t} = \mu + timeF + compF + f(pe_{2i},t) + f(a_{2i},t) + \varepsilon_{i,t}$

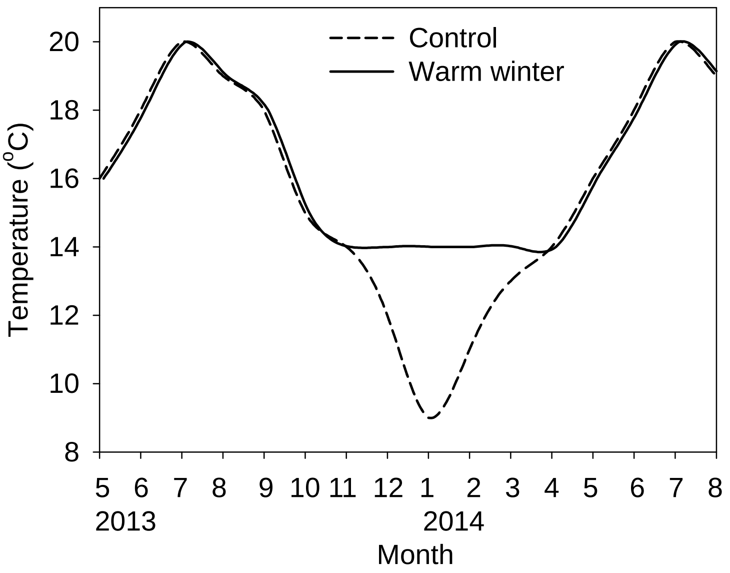

Fig. S1. Seasonal change in the water temperature of the F1 growth tanks of normal and warm winter treatment groups. The temperature in the normal winter group is similar to the seasonal variation in water temperature in the Rio Ulla (Source: Augas de Galicia, Xunta de Galicia), from which the F0 fish were collected.

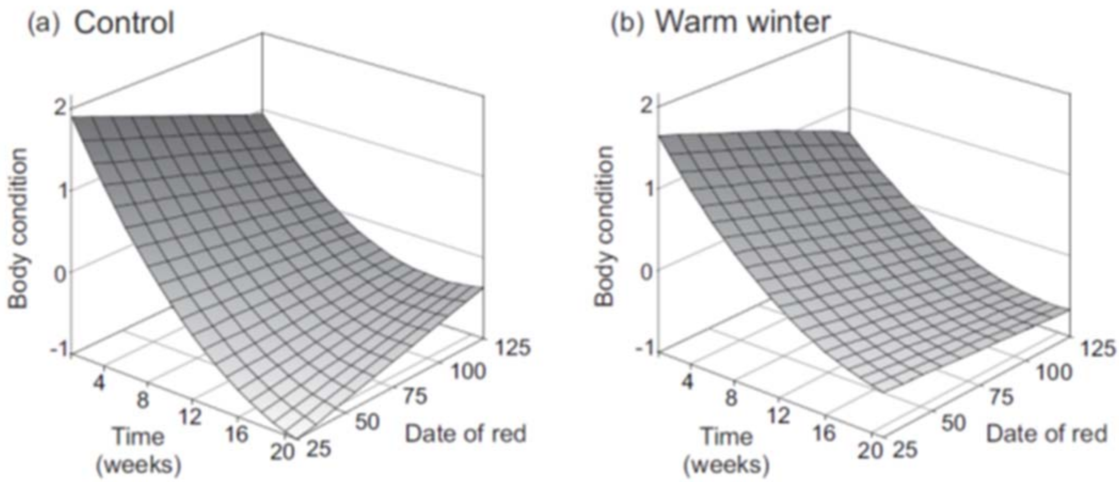

Fig. S2. Relationship between seasonal change in body condition and time of maturation (Julian date, 1 = 1 January 2014) of males from the (a) control and (b) warm winter groups.

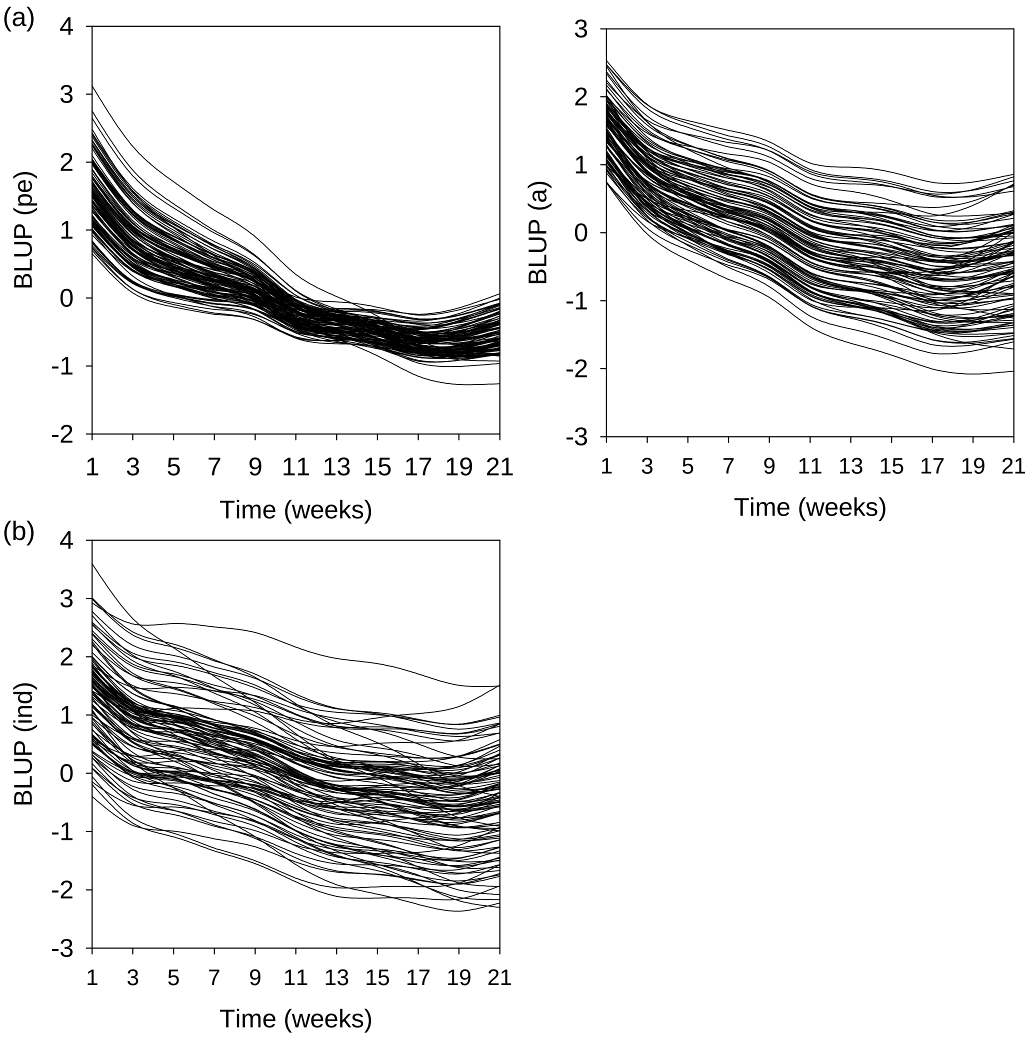

Fig. S3. Plots of the reaction norms for body condition of individual sticklebacks of the (a) control and (b) warm winter schemes based on the fixed effects of elevation and slope and the BLUP values from the best fit models presented in table S1. BLUPs are printed at the permanent environment (*pe*) and additive genetic (*a*) level in the controls and at the individual (*ind*) level in the warm-treated individuals in which the decomposition of genetic and permanent environment components did not significantly improve the model.
